# Epiblast-specific *Snai1 *deletion results in embryonic lethality due to multiple vascular defects

**DOI:** 10.1186/1756-0500-2-22

**Published:** 2009-02-06

**Authors:** Hilda Lomelí, Christa Starling, Thomas Gridley

**Affiliations:** 1 Departamento de Genética del Desarrollo y Fisiología Molecular, Instituto de Biotecnología, Universidad Nacional Autónoma de México, Cuernavaca, Morelos, México; 2 The Jackson Laboratory, Bar Harbor, ME 04609, USA

## Abstract

**Background:**

Members of the Snail gene family, which encode zinc finger proteins that function as transcriptional repressors, play essential roles during embryonic development in vertebrates. Mouse embryos with conditional deletion of the Snail1 (*Snai1*) gene in the epiblast, but not in most extraembryonic membranes, exhibit defects in left-right asymmetry specification and migration of mesoderm cells through the posterior primitive streak. Here we describe phenotypic defects that result in death of the mutant embryos by 9.5 days of gestation.

**Findings:**

Endothelial cells differentiated in epiblast-specific *Snai1*-deficient embryos, but formation of an interconnected vascular network was abnormal. To determine whether the observed vascular defects were dependent on disruption of blood flow, we analyzed vascular remodeling in cultured allantois explants from the mutant embryos. Similar vascular defects were observed in the mutant allantois explants.

**Conclusion:**

These studies demonstrate that lethality in the *Snai1*-conditional mutant embryos is caused by multiple defects in the cardiovascular system.

## Background

In mammals, there are three Snail family genes: *Snai1 *(formerly *Snail*), *Snai2 *(formerly *Slug*), and *Snai3 *(reviewed in [1,2]). We have shown that mouse embryos homozygous for a *Snai1 *null mutation (*Snai1*^-/- ^embryos) exhibit defects in mesoderm formation and die shortly after embryonic day (E) 7.5 [3], likely due to defects in the extraembryonic membranes. In contrast, mouse embryos (*Meox2-Cre; Snai1*^*flox*/- ^embryos) with deletion of the *Snai1 *gene specifically in the epiblast (i.e., the embryo proper plus extraembryonic mesoderm) survive past the period of lethality at E7.5. These embryos exhibit defects in left-right asymmetry specification and delayed progression of mesoderm cells through the posterior primitive streak [4]. Here we describe phenotypic defects in *Meox2-Cre; Snai1*^*flox*/- ^(hereafter designated *Snai1-cko*) embryos that result in death of the mutant embryos by approximately E9-E10.

## Methods

### Mice

The targeted null allele of the *Snai1 *gene [3] and the *Snai1*^*flox *^conditional allele [5] have been described. *Snail1*^*flox*/*flox *^mice were maintained as homozygotes. *Meox2-Cre *mice [6] were obtained from the Jackson Laboratory. For the experiments described here, male mice heterozygous for both the *Meox2-Cre *allele and the *Snai1 *null allele (*Snai1*^+/-^) were crossed to *Snai1*^*flox*/*flox *^females, and embryos were isolated at E8.5 and E9.5. Embryos of the genotype *Meox2-Cre/+; Snai1*^*flox*/- ^(referred to as *Snai1-cko*, for *Snai1 *conditional knockout) were analyzed. Littermate embryos lacking one or more of the following alleles (*Meox2-Cre*, *Snai1*^*flox *^or *Snai1 *null) were used as controls. Embryos were genotyped by PCR of DNA isolated from the yolk sac. All animal experiments were performed under a protocol approved by the Jackson Laboratory Animal Care and Use Committee.

### Allantois culture

The allantois was dissected from E8.5 mouse embryos using tungsten needles, and was placed individually on collagen or fibronectin-coated coverslips in 8-well culture dishes (BD Biocoat). Explants were cultured in 0.5 ml of culture medium (DMEM 4.5 g/l glucose, 10 mM L-glutamine, Pen-Strep), containing 15% fetal calf serum for 18 hours. Explants then were washed and fixed in 4% paraformaldehyde or Methanol:DMSO (4:1) for 20 min at room temperature and processed for immunohistochemistry or TUNEL assay. For morphometric analysis of the allantois cultures, cultures were fixed as above, immunostained for PECAM-1 expression, and counterstained with eosin. The diameter of the vascular network, as defined by the extent of PECAM-1 positive vessels, was measured [7]. The diameter of the underlying layer of eosin-stained mesothelial cells was also measured. Values were expressed as mean ± standard deviation. Differences between the means of the mutants and controls were tested for statistical significance using the Unpaired two-tailed Student's *t *test. *P *values < 0.05 were considered to be statistically significant.

### Immunostaining and TUNEL analysis

For immunohistochemistry on explant cultures, fixed cells were permeabilized and blocked in 0.1% Triton X-100/10% goat serum/PBS for 30 minutes and incubated for one hour with a 1:50 dilution of the corresponding primary antibody. Antibodies included rat-monoclonal anti-mouse CD31 (PECAM-1) (BD Biosciences Pharmingen), mouse monoclonal anti-VCAM-1 (eBioscience) and anti-mouse CD144 (VE-cadherin) (BD Biosciences Pharmingen). Explants were washed and incubated for one hour in the corresponding secondary antibody. Horseradish peroxidase-coupled secondary antibodies were from Jackson ImmunoResearch. Eosin counterstaining was done after DAB (Diamino benzidine tetrahydrochloride) color reaction. For immunofluorescence, an Alexa Fluor 488-labeled secondary antibody (Invitrogen) was used, and slides were mounted with DAPI (4'-6-Diamidino-2-phenylindole). For TUNEL analysis, the *In Situ *Cell Death Detection Kit, Fluorescein (Roche Applied Science) was used according to the manufacturer's instructions. Optical sectioning of entire allantois explants was performed by confocal microscopy. The complete *Z *series was then collapsed and fluorescent cells per field were counted (*n *= 3). Results are presented as the mean ± sem. Statistical significance was determined using the Paired two-tailed Student's *t *test, with *P *values < 0.05 considered to be statistically significant.

## Results

### Vascular defects in mouse embryos with epiblast-specific deletion of the Snai1 gene

**Figure 1:**
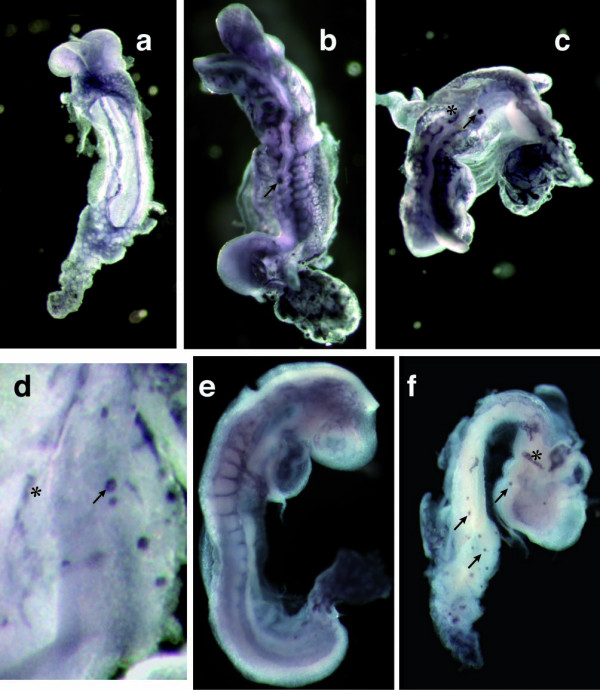
**Vascular defects in *Snai1-cko *embryos**. (a-d) PECAM-1 immunostained E8.5 littermate control (a) and *Snai1-cko *(b-d) embryos. (d) Higher magnification view of the dorsal region of a different embryo showing endothelial cell aggregates (arrow). (e, f) VE-cadherin immunostained E8.5 control (e) and *Snai1-cko *(f) embryos. Asterisks indicate discontinuous vessels, and arrows point to isolated aggregates of PECAM-1 positive cells.

No *Snai1 *transcripts can be detected in *Snai1-cko *embryos or allantois by E8.0 [4]. We visualized the vascular network of *Snai1-cko *mutant embryos and littermate controls by staining with a monoclonal antibody to platelet endothelial cell adhesion molecule-1 (PECAM-1), a vascular endothelial cell marker [8]. At E8.5, PECAM-1 positive cells were present in the *Snai1-cko *embryos (Fig. 1b–d), confirming the differentiation of endothelial cells in these embryos. Vascular defects in *Snai1-cko *embryos were completely penetrant (*n *> 8). A primitive vascular network forms in most *Snai1-cko *embryos, but many of these vessels were discontinuous. The *Snai1-cko *embryos also exhibited unusual aggregations of PECAM-1 positive cells (Fig. 1b–d). Both the discontinuous vessels and the endothelial cell aggregates were also detected in *Snai1-cko *embryos immunostained for VE-cadherin (Cdh5) (Fig. 1e, f), another endothelial cell marker [9]. These results indicated that although mesoderm cells from *Snai1-cko *embryos can differentiate into endothelial cells, *Snai1 *function in the embryo is required for the proper morphogenesis of endothelial cells into a primitive capillary plexus and for its subsequent growth and remodeling. However, these results do not establish whether *Snai1 *gene function is required autonomously in the endothelial cells, or nonautonomously in surrounding mesoderm cells that also express high levels of *Snai1 *transcripts at these developmental stages [10].

### *Snai1-cko *allantois explants exhibit abnormal vascular morphogenesis

Our previous study of *Snai1-cko *embryos had demonstrated aberrant heart looping as the result of defects in left-right asymmetry specification [4], which likely affects blood flow in the mutant embryos. Since alterations in blood flow can cause defects in vascular development and remodeling [11], we assessed vascular development and remodeling in *Snai1-cko *embryos in a situation that is not dependent on blood flow. The embryonic allantois is a widely utilized model system for the study of early vascular development in mice (reviewed in [12]). The allantois contains only three known cell types (endothelial cells, mesothelium and mesenchyme of the allantoic core), and allantois cultures have been validated by several groups as a powerful model for the study of the mechanisms of blood vessel formation and remodeling [13-17].

**Figure 2:**
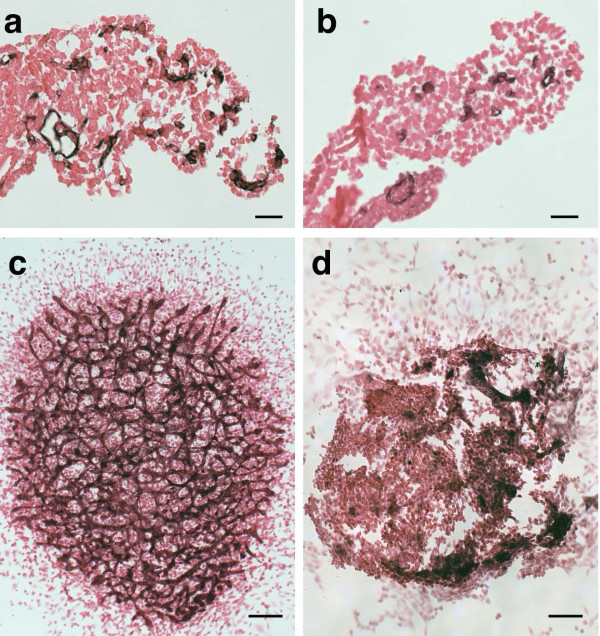
**Vascular morphogenesis in *Snai1-cko *allantois and allantois explants plated on collagen**. (a, b) Histological sections of PECAM-1 immunostained E8.5 littermate control (a) and *Snai1-cko *(b) allantois. Vascular endothelial cells are present in both control and *Snai1-cko *allantois in vivo. (c, d) PECAM-1 immunostained allantois explants derived from E8.5 control (c) and *Snai1-cko *(d) allantoises, grown on collagen. *Snai1-cko *explants are less expanded than control littermate explants, and instead of the interconnected vascular network that can be seen in the littermate control explant, clusters of PECAM-1 positive cells that do not form a network are present in the *Snai1-cko *explant. Scale bar in (a, b): 20 μm; (c, d) 100 μm.

To evaluate whether vascular defects observed in *Snai1-cko *embryos may be secondary to alterations in blood flow, we set up allantois explant cultures, in which no blood flow occurs. Since *Snai1-cko *embryos at E8.5 exhibited a shorter, bulbous allantois, before turning to the explant culture model we sectioned PECAM-1 immunostained allantois from *Snai1-cko *embryos. This analysis confirmed that endothelial cells were present in the *Snai1-cko *allantois (Fig. 2b). We next cultured E8.5 allantois explants on collagen-coated plastic wells. After 18 hours of incubation, control explants exhibited a network of PECAM-1 positive capillary-like vessels (Fig. 2c). In contrast, allantois explants from *Snai1-cko *embryos exhibited only clusters of PECAM-1 positive endothelial cells that failed to form a vascular network (Fig. 2d).

**Figure 3:**
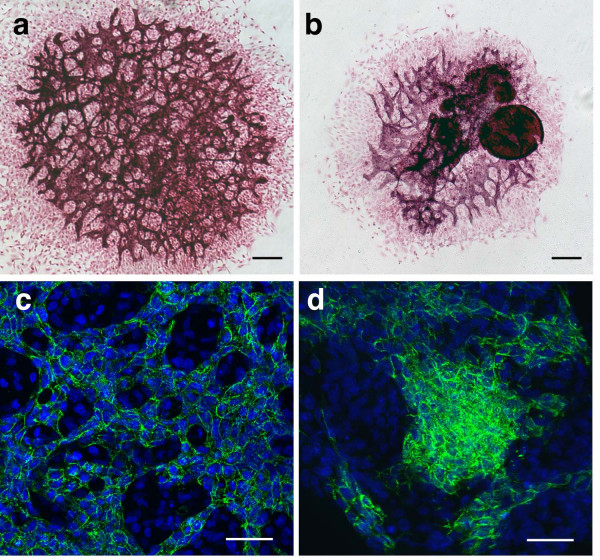
**Fibronectin stimulates formation of a vascular plexus in the *Snai1-cko *allantois explants**. (a, b) PECAM-1 immunostained allantois explants derived from E8.5 littermate control (a) and *Snai1-cko *(b) allantoises grown on fibronectin. Although morphologically abnormal, formation of a vascular network can be observed in the *Snai1-cko *explant. *Snai1-cko *explants also exhibited PECAM-1 positive clusters of cells that did not adhere to the plate but grew up into the culture media. (c, d) Confocal imaging of DAPI (blue) and PECAM-1 (green) immunofluorescent allantois explants from E8.5 control (c) and *Snai1-cko *(d) allantoises. The *Snai1-cko *explant exhibits an intense aggregation of endothelial cells in one region of the explant, and the absence of connections between endothelial cells in other regions. Scale bar in (a, b): 100 μm; (c, d) 50 μm.

**Figure 4:**
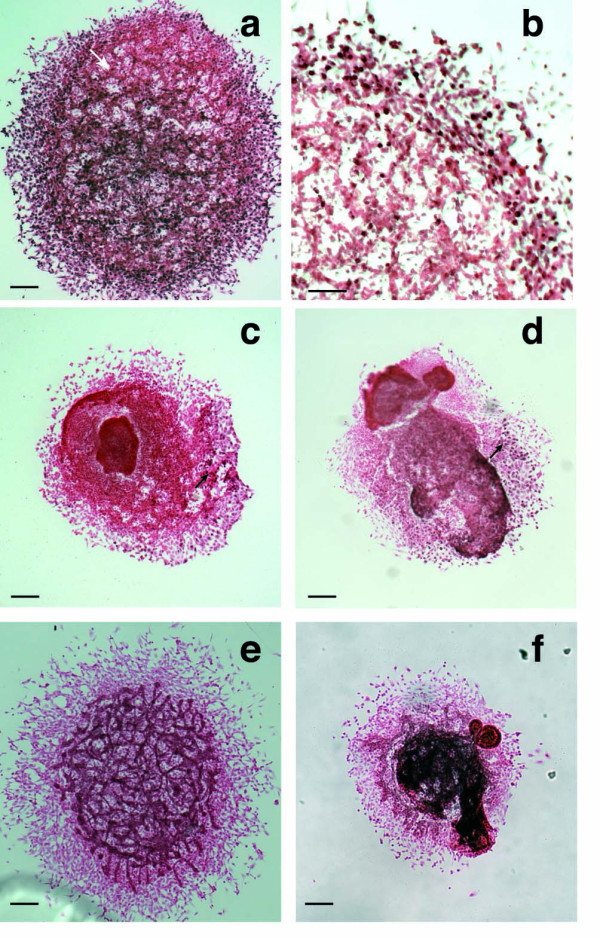
**VCAM1 and VE-cadherin expression in control and *Snai1-cko *allantois explants**. (a-d) VCAM1-immunostained allantois explants derived from littermate control (a, b) and *Snai1-cko *(c, d) embryos plated on fibronectin. (b) Higher magnification of a control explant showing that VCAM positive cells are more abundant in the periphery of the explant. In *Snai1-cko *mutant explants, fewer VCAM1-positive cells are observed, but these cells also tend to localize to the periphery of the explant. (e, f) VE-cadherin-immunostained allantois explants derived from E8.5 control (e) and *Snai1-cko *(f) embryos. Scale bar (a, c-f): 100 μm, (b) 50 μm.

To study the response of the *Snai1-cko *mutant allantois explants to other mediators of extracellular matrix adhesion and to optimize culture conditions, we plated the allantois explants on other substrates. When plated on fibronectin-coated wells, *Snai1-cko *allantois explants formed a PECAM-1 positive network of endothelial cells, but obvious morphological defects were evident in the mutant explants (Fig. 3b, d). *Snai1-cko *explants exhibited the accumulation of PECAM-1 positive clusters of cells that did not completely adhere to the plate but grew up into the culture media (Fig. 3b). Confocal images of PECAM-1 immunofluorescent allantois cultures revealed that control littermate allantois explants formed an interconnected anastomosing vascular network (Fig. 3c). In contrast, the network formed by the *Snai1-cko *allantois explants exhibited an irregular aggregation of endothelial cells with PECAM-1 positive cells concentrated in certain regions but absent from other large regions (Fig. 3d). To determine whether mesothelial cells differentiated in the allantoic explants, we assessed VCAM-1 expression. In E8.5 control explant cultures, VCAM-1 positive cells were enriched in the periphery of the explant culture, and less abundantly on top of the vascular plexus (Fig. 4b). In contrast, in allantoic explants from *Snai1-cko *mutant embryos, fewer VCAM-1 positive cells were detected (Fig. 4c, d), but these were also enriched in the periphery. VE-cadherin immunostaining of control littermate allantois explants revealed formation of an interconnected vascular network (Fig. 4e) similar that that revealed by PECAM-1 staining (Fig. 3a). VE-cadherin immunostaining of *Snai1-cko *allantois explants revealed the presence of VE-cadherin-positive cells, but these cells did not form a morphologically normal vascular network (Fig. 4f).

We also noted that expansion of the allantois explants was reduced in *Snai1-cko *mutants. To quantify this observation, we measured the diameters of the vascular networks in E8.5 allantois explants cultured on fibronectin [7]. The average diameter of the vascular networks formed by littermate control explants was 1.35 mm ± 0.15 (*n *= 12), which was 1.4 fold greater than the average diameter exhibited by *Snai1-cko *explants (0.99 mm ± 0.18; *n *= 9; *P *< 0.05). We also measured expansion of the mesothelial layer formed in these explants. The average diameter of the mesothelial discs formed by control allantois explants also was about 1.4 fold greater than those formed in *Snai1-cko *explants (1.93 mm ± 0.27 for the control explants versus 1.39 mm ± 0.24 for the mutant explants; *P *< 0.05).

### Apoptosis is increased in *Snai1-cko *allantois explants

**Figure 5:**
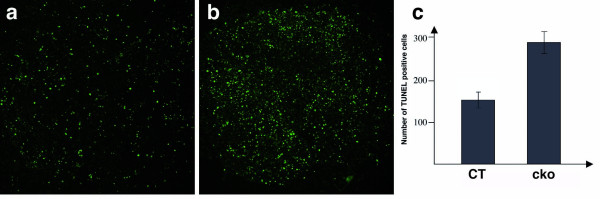
**Increased apoptosis in *Snai1-cko *allantois explants**. (a, b) Fluorescent TUNEL staining of explants derived from E8.5 control and *Snai1-cko *allantois. (c) Histogram representing numbers of TUNEL-positive cells from three different explant cultures for each genotype. *P *< 0.05. CT: control; cko: *Snai1-cko*.

The *Snai1 *and *Snai2 *genes have a demonstrated role in the protection of cells from apoptotic cell death [18-23]. We assessed apoptotic cell death in allantois explants by TUNEL assay. *Snai1-cko *explants exhibited an obvious increase in the number of fluorescent cells (Fig. 5b). To quantify this observation, TUNEL-positive fluorescent cells were counted in one field of a complete *Z *series collapsed from confocal images. In littermate control explants, a mean of 161 ± 18 (*n *= 3) TUNEL-positive cells per field were present, while in the *Snai1-cko *explants a mean of 284 ± 25 (*n *= 3) TUNEL-positive cells per field were present (Fig. 5c). This difference represented a 76% increase of TUNEL-positive cells in the *Snai1-cko *mutant explants compared to the controls (*P *< 0.05). Further work will be required to determine the mechanism by which loss of *Snai1 *function in the allantois cultures from *Snai1-cko *embryos leads to an increase in cell death.

## Discussion

Taken together, our results demonstrate that the cause of death of *Snai1-cko *embryos at E9-E10 is multiple cardiovascular defects (i.e., heart looping defects, vascular morphogenesis and remodeling defects, and failure of allantois-chorion fusion). In *Snai1-cko *embryos, angioblast differentiation into endothelial cells occurred, but morphogenesis into an interconnected vascular network was defective. The observation of vascular defects in the allantois cultures, in which no circulation occurs, demonstrates that at least some of the vascular defects observed in *Snai1-cko *embryos are not secondary to defects in blood flow. Vascular network formation by *Snai1-cko *allantois cultures was better on fibronectin than on collagen, although network formation was much worse than that of littermate control cultures on both substrates. Recent work has demonstrated that *Snai1 *over-expression in epithelial cell lines can regulate expression of integrins and laminins [24]. *Snai1 *over-expression also enhanced the ability of these cells to attach to a fibronectin-coated substratum. These results are consistent with our finding that *Snai1-cko *cells do not adhere as well as control littermate cells to fibronectin (Fig. 3).

We do not know at present whether *Snai1 *function is required autonomously within endothelial cells, or is required nonautonomously in the surrounding tissues. Due to the strong and widespread expression of *Snai1 *RNA during early mouse embryogenesis [10], plus the lack of a good anti-SNAI1 antibody, we have not been able to determine unequivocally whether the *Snai1 *gene is expressed in endothelial cells in vivo during early stages of postimplantation mouse development (e.g., days E8-E10 of gestation). However, the *Snai1 *gene is expressed at similar stages in endocardial cells of the heart [25], which are functionally similar to endothelial cells. SNAI1 protein also is expressed in human umbilical vein endothelial cells [26], and *Snai1 *RNA is expressed in endothelial cells purified from differentiated mouse embryonic stem cells [26]. These data suggest the likely possibility that *Snai1 *gene function is required autonomously in endothelial cells.

## Competing interests

The authors declare that they have no competing interests.

## Authors' contributions

HL participated in study concept and design, carried out the experiments, performed data analysis and prepared the manuscript figures. CS helped carry out the histological and immunohistochemical analyses. TG participated in study design and data analysis. HL and TG wrote the manuscript. All authors read and approved the final manuscript.
